# In-vitro scientific validation of anti-inflammatory activity of *Punica granatum L.* on Leukemia monocytic cell line

**DOI:** 10.4314/ahs.v24i4.31

**Published:** 2024-12

**Authors:** Sharmistha Dutta, Khushbu Nishad, Talambedu Usha, Nijalingappa Ramesh, Sushil Kumar Middha

**Affiliations:** 1 Department, Biotechnology, School of Applied Sciences Reva University, Rukmini Knowledge Park, Bengaluru-560 064, India; 2 Department of Biochemistry, Maharani Lakshmi Ammanni College For Women, Bengaluru-560 012, India; 3 Department of Biotechnology, Maharani Lakshmi Ammanni College For Women, Bengaluru-560 012, India

**Keywords:** *Punica granatum L*, Monocytic leukaemia cell line, cytotoxicity, anti-inflammation

## Abstract

**Background:**

The induction of the inflammatory cascade results in the production of a number of inflammatory mediators, including prostaglandin E2 (PGE2), nitric oxide (NO), and proinflammatory cytokines like TNF-, IL-, and IL-6. This study examined the cytotoxicity and anti-inflammatory properties of a methanolic crude extract of Punica granatum L. peel (PPM) on monocytic leukaemia cell line (THP-1).

**Materials and methods:**

The PPM along with Quercetin as reference was used to assess the cytotoxic effect on THP-1 cells and describe its effect on pro-inflammatory cytokines such as COX-2, TNF-α, IL-6 against cancer cell line by flow cytometry.

**Results:**

The percentage of viable cells significantly decreased which correlates to non-toxicity whereas quercetin was found to be highly toxic, the IC50 could not be calculated because of drug precipitation. There was a significant decrease in the expressions of inflammatory cytokines upon pre-treatment of the cells with PPM prior to LPS stimulation.

**Conclusion:**

Our findings indicate that no cytotoxicity was observed after the treatment of THP-1 cells with PPM (25-400 µg/ml), but at higher concentration (400µg/ml), the cell viability decreased to 84% and attenuated the expression level of inflammatory cytokines. The inhibitory effect of the extract on pro-inflammatory factors production may provide a theoretical source on upcoming treatment of inflammation.

## Introduction

Pomegranate (*Punica granatum L*.), is a deciduous therapeutic plant since old times, and is likewise considered as a mysterious plant^1^. It is fruit-bearing shrub which originates from Middle East and India^2^. Traditionally, pomegranate has all around reported appreciation in many cultures as an image of longevity and well being^3^. All parts of the tree, including peel, have specific applications in traditional medicinal systems such as Chinese, Ayurvedic and Unani^4^. For instance, it is used as a solution for different neurotic conditions such as malaria, dental plaque, diabetes, dysentery and intestinal infections^5^, ^6^. The fruit rind and the bark of the pomegranate tree have been utilized for the treatment of dysentery, digestive parasites and diarrhea. Scientific information reveals that antioxidant, anti-inflammatory and anti-cancer activity of pomegranate can be to a great extent ascribed to its high substance of polyphenols. Pomegranate peel (PP) is significant natural source of phenolics such as flavonoids, proanthocyanidins and ellagitannins^7^.

Lansky and Newman^8^ concluded that the extracts of pomegranate shows medical applications in various diseases where inflammation is believed to play a fundamental etiology, such as cancer. Inflammation represents the first defensive barrier and occurs through two steps, acute and chronic^9^. Chronic inflammation is concerned with the beginning of few provocative problems and inflammatory disorders such as inflammatory bowel disease, rheumatoid arthritis and chronic obstructive pulmonary disease^10^. Pomegranate and its significant parts have been generally shown to have anti-inflammatory properties. For example, cold pressed PG seed oil has been known to possess anti-inflammatory activity since it repressed in vitro both cyclooxygenase and lipoxygenase enzymes. As per the literature data, few examinations explored the effects of different fractions extracted from pomegranate on various malignant cell lines. It has been shown that PP extract reduces proliferation and prompts apoptosis in MCF-7 cells depending on concentration applied and incubation time^11^. It was demonstrated that pomegranate peel extract reduced the production of reactive oxygen species (ROS), inflammatory cytokines expression and prevented other inflammatory events due to particulate matter^12^. Study demonstrated that using higher concentration of pomegranate peel extract, anti-inflammatory effects were strongly exhibited in both in vivo and ex vivo models by the repression of CXCL8 concentrations in cell and tissue supernatants, respectively^13^. Research studies demonstrated that the treatment with pomegranate polyphenols significantly diminished the release of two inflammatory promoting compounds such as nitric oxide (iNOS) and cyclooxygenase (COX-2), that play a very vital role in inflammation^14^.

Recently, Pepe et al.,(2020)^15^ evaluated that the antioxidant and anti-inflammatory capacity of pomegranate extract reduced the oxidative stress and increase in the levels of cytoprotective enzymes, such as heme oxygenase 1 (HO-1) and NAD(P)H dehydrogenase quinone 1 (NQO-1), and tight junction proteins. The nuclear factor-kappa B (NF-κB) pathway has been identified as a key mediator of inflammation and serves as an important target for drug development^16^. ROS is thought to be involved in inflammatory gene expression through the redox-based activation of the NF-κB signaling pathway. It has been shown that many antioxidants inhibited inflammatory gene expression and NO production by suppressing NF-κB activation through the removal of ROS.Pomegranate peel has been reported in the past by our lab for its anti-diabetic properties^7^. We also sequenced and annotated the genome of the superfood Indian Punica granatum to explore the PPP pathway^6^. Pomegranate peel polyphenols and their components have been shown to significantly inhibit the expression of pro-inflammatory mediators and the molecular mechanism was associated with the inhibition of MAPKs activation^17^, ^18^. The aim of this work was to examine the cytotoxicity and inflammatory effects of pomegranate peel extract (PPM) on Leukemia monocytic cell line.

## Materials and Methods

### Chemicals

The quercetin reference standard, RPMI-1640 medium, Foetal Bovine Serum, penicillin was purchased from Sigma-Aldrich (USA). The cell lines (THP-1) were purchased from NCL, Pune.

### Collection and preparation of *P. granatum* peels extract

Fruits of Punica granatum were purchased from the local markets of Bengaluru, India. The fruits were cleaned under running tap water, and the fruit peels were gently removed and allowed to dry in shade. The dried material was finely powdered using a mixer grinder.. 50g of the powdered pomegranate peel was weighed extracted thrice using the Soxhlet apparatus at 40-60°C using 70% methanol. After three cycles, the filtrate was collected and dried at 55°C using water bath^19^, ^20^. The pomegranate peel crude methanolic extract PPM prepared was used for further investigations..

### Cell culture

Using Roswell Park Memorial Institute (RPMI-1640) medium supplemented with 10% Foetal Bovine Serum (FBS), 100 U/ml of penicillin, and at 37°C in a humidified atmosphere with 5% CO2,THP-1 a monocytic leukemia cell line as grown in T-25 flasks.

### Measurement of Cytotoxicity of PPM

Cells cultured in T-25 flasks were aspirated into a 5mL centrifuge tube and centrifuged at 300*g. The cell count of the obtained pellet was adjusted, using DMEM HG medium, such that 50µl of suspension contained approximately 10,000 cells. Followed by it 50µl of the cell suspension was added to each well of the 96 well microtitre plate, and was incubated at 37°C and 5% CO_2_ atmosphere for 24 h. 50µl different concentrations of of PPM (25, 50, 100, 200, 400µg/ml) quercetin (25, 50, 100, 200, 400µg/ml) and Cisplatin (6.25,12.5,25,50 and 100 µg/ml), were added to the respective wells. The plate was then incubated at 37°C and 5% CO_2_ atmosphere for 24 h. 100µl of medium containing MTT reagent was then added to each well to get a final concentration of 0.5mg/mL and the plate was incubated at 37°C and 5% CO_2_ atmosphere for 3 h. 100µl of Dimethyl sulfoxide (DMSO) was added to the wells to dissolve the generated formazan. The absorbance was measured using an ELISA microplate photometer (Multiskan™ FC, Thermo Scientific™), at 570 and 630nm. After removing the background and the blank, the percentage of growth inhibition (IC50) was computed, and the dose-response curve for the cell line was used to generate the concentration of PPM required to inhibit cell growth by 50%.

Measurement of COX-2, IL-6 and TNF-α production THP-1 cells were cultured in 6-well plate at a density of 3 x 105 cells/2 mL and incubated in a CO2 incubator overnight at 37°C for 24 hours for studying the expression of COX-2, TNF- α and IL-6. The cells were treated with PPM(25µg/ml),quercetin (12.5 µg/ml) and incubated for 24 hours at 37°C. The cells were stimulated with LPS(2 µg/ml) leaving the untreated cells.1µl of protein transport inhibitor (BD GolgiStopTM) was added to each well that aids in the accumulation of expressed cytokines within the cells. After 24 hours treatment the cells were harvested and centrifuged for 5 minutes at 300*g at 25µC and washed twice with PBS.. 0.5ml of 2% paraformaldehyde solution was added and incubated for 20 minutes. Followed by it a wash with 0.5% bovine serum albumin(BSA) was carried out PE Anti-Human IL-6 monoclonal antibody (Catalog No. 12-7069-81 Thermofisher, USA), FITC Mouse Anti-Human TNF, Catalog No. 562082, PE Mouse Anti-Human COX-2, Catalog No. 565125, BD Biosciences was added to a final volume of 100µL of cell suspension in three different plates respectively and incubated for 30 minutes in the dark after at room temperature. Again, the cells washed with 0.5BSA. Finally, 0.5ml of PBS was added and the changes in IL-6, TNF and COX2 expression was detected by measuring the cells positive in PE fluorescence in FL2 detector using a 575nm band pass filter, FITC fluorescence in FL1 detector using a 525nm band pass filter of cytomics FC500,Beckman coulter, USA.

### Statistical analysis

All experiments were carried out in triplicates and the results are presented as mean± standard deviation. The statistical analysis of variance (ANOVA) was carried out using Microsoft office Excel 10 and all the graphs were plotted using the same. p< 0.05 was considered as statistical significance

## Results and Discussion

**Figure 1:**
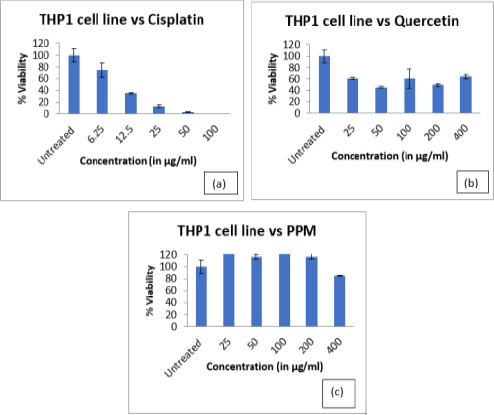
Cytotoxic activity of different concentration of PPM in THP-1 cells as determined by MTT reduction assay. Cell viabilities are presented as percentages of viable cells per total cells

**Figure 2:**
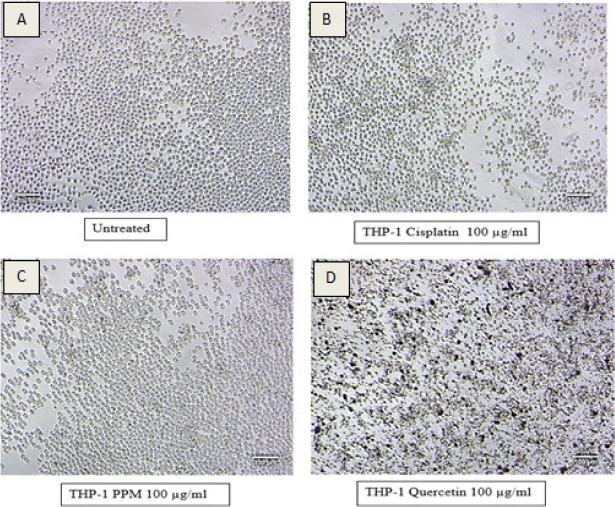
Morphological changes of THP-1 cells after 24hrs of treatment with Cisplatin, PPM and Quercetin during MTT Assay

In this study, we examined the cytotoxicity and proinflammatory effect of PPM. Figure 1(a) shows the percentage of cell viability of cells treated with cisplatin. The number of viable cells decreased from 0.5%-74.5%-0.05% with an increase in cisplatin concentration from 6.25 to 100 µg/ml. The cell viability in PPM treatment also significantly decreased, however the percentage viability is slightly higher than the untreated cells in cells treated with 25 to 100 µg/ml of PPM, which correlates to non-toxicity (Figure 1(C)). MTT assay results for quercetin were found to be highly toxic, not proportional to the concentration (Figure 1(b)) and the IC50 could not be calculated due to high absorbance. The high absorbance can be attributed to drug precipitation on the plate at higher concentrations as shown in Fig 2(D). Figure 2 represents the morphological changes noted in THP-1 cells, after 24 hours of treatment. It is noted that the morphology was similar in both untreated and cells treated with PPM indicating no toxicity of PPM (Figure 2a and C). Less viable cells can be clearly noted in cells treated with cisplatin. Many dead cells with precipitation of quercetin is seen cells treated with quercetin. The IC50 of Cisplatin was found to be 9.46±3.49 µg/ml and PPM was found to be non-toxic to cells.

### COX-2 Expression

**Fig 3:**
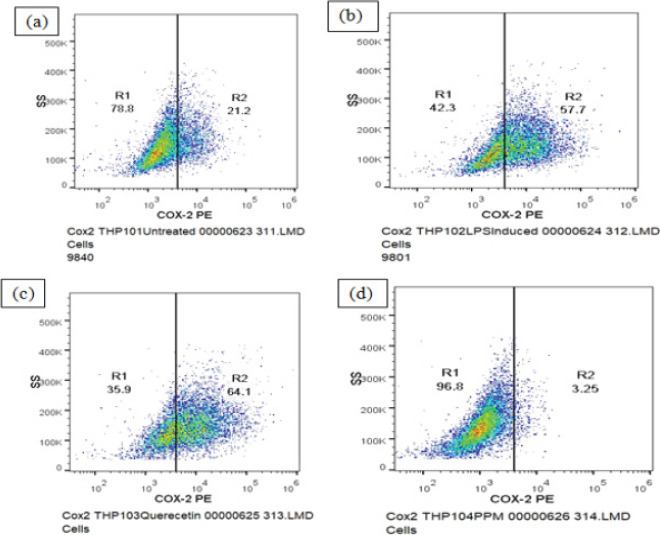
DOT PLOT showing Methanol extract of pomegranate peel (PPM) inhibits the expression of inflammatory cytokine COX2. (a) Untreated THP-1 cells (b) LPS induced THP-1 cells (c) THP-1 cells treated with quercetin (d) THP-1 cells treated with PPM

The expression of COX2 in THP-1 cells in each group was tested. COX2 expression increased after the cells were stimulated with LPS. The percentage of cells with low COX2 and high levels of COX2 was found to be 78.8±1.36and 21.20±4.53 respectively and geometric mean fluorescence intensity (MFI) of PE COX-2 was found to be 5258±18.93. A decrease in COX-2 expression was observed upon pre-treatment of the cells with sample PPM prior to LPS treatment as compared to LPS induced control cells whereas, significant decrease was seen in the mean fluorescence intensity of PE fluorochrome and COX-2 high cells (Figure 3). Decrease in the expression of COX-2 was not observed upon pre-treatment with quercetin (Table 2).

**Table 2:** Effect of Methanol extract of Pomegranate peel on COX-2 Expression

Sample Name	Geometric mean fluorescence intensity (MFI) of PE COX-2 (FL2-A parameter)	% of Cells COX-2 Low	% of Cells COX-2 High
**Untreated**	2260±41.63	78.8±1.36	21.20±4.53
**LPS induced - 2 µg/ml**	5258±18.93	42.3±3.82	57.70±6.98
**Quercetin - 12.5 µg/ml**	6365±34.29	35.9±1.94	64.10±5.91
**PPM - 25 µg/ml**	1053±8.92	96.8±7.64	3.25±0.82

### TNF-α Expression

**Fig 4:**
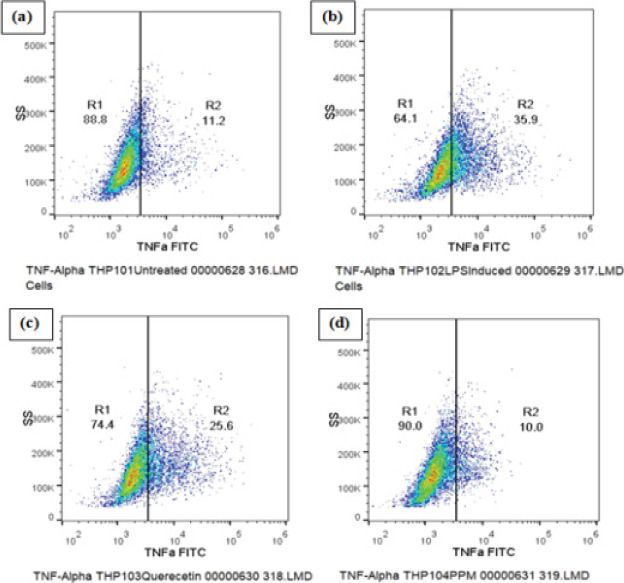
DOT PLOT showing Methanol extract of pomegranate peel (PPM) and Quercetin reduces the expression of inflammatory cytokines TNF-α.(a) Untreated THP-1 cells (b) LPS induced THP-1 cells (c) THP-1 cells treated with quercetin (d) THP-1 cells treated with PPM

The expression of pro-infammatory cytokine TNF-α was analysed by Flow cytometric method. The results show significant decrease in TNFα expression in the cells pre treated with PPM before LPS stimulation as compared to LPS induced control cells (Figure 4). quercetin (2724±17.93) and PPM(1597±10.64) significantly decreased the production of TNFα, observed as a decrease in the mean fluorescence intensity of FITC fluorochrome compared to 3129±18.74 MIF of LPS treated cells and decrease in the percentage of TNFα high cells as shown in Table 3 and Figure 4.

**Table 3:** Effects of Methanol extract of Pomegranate peel on TNF- α Expression

Sample Name	Geometric mean fluorescence intensity (MFI) of PE TNFα (FL1-A parameter)	% of Cells TNFα Low	% of Cells TNFα High
**Untreated**	2064±29.91	88.8±10.92	11.2±1.76
**LPS induced - 2 µg/ml**	3129±18.74	64.1±5.81	35.9±7.61
**Quercetin - 12.5 µg/ml**	2724±17.93	74.4±9.27	25.6±6.10
**PPM - 25 µg/ml**	1597±10.64	90.0±8.91*	10.0±2.98*

* *indicated the significance level at p<0.5 as compared with LPS induced cells*

### IL-6 Expression

**Fig 5:**
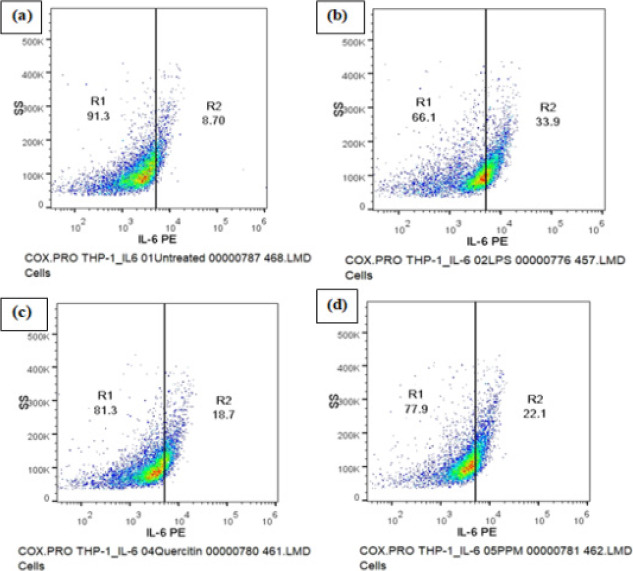
DOT PLOT showing the effect of Methanol extract of pomegranate peel (PPM) and Quercetin on the expression of inflammatory cytokine IL-6. A decrease in IL-6 expression was observed upon pre-treatment of the cells with Sample PPM and Quercetin prior to LPS stimulation. (a) Untreated cells (b) LPS induced cells (c) Cells treated with quercetin (d) Cells treated with PPM

A decrease in IL-6 expression was observed upon pre-treatment of the cells with sample PPM and quercetin prior to LPS stimulation as compared to LPS induced control cells where significant decrease was seen in the percentage of IL-6 high cells (Table 4). The changes in IL-6 expression were detected by measuring the cells positive in PE (yellow) fluorescence in the FL2 detector (Figure 5).

**Table 4:** Effect of Methanol extract of Pomegranate peel on IL-6 Expression

Sample Name	Geometric mean fluorescence intensity (MFI) of PE IL-6 (FL2-A parameter)	% of Cells IL-6 Low	% of Cells IL-6 High
**Untreated**	1883±19.12	91.30±11.81	8.70±3.13
**LPS induced - 2 µg/ml**	2842±31.91	66.10±2.74	33.90±1.72
**Quercetin - 12.5 µg/ml**	2405±8.71	81.30±3.81	18.70±2.68
**PPM - 25 µg/ml**	2969±6.02	77.90±6.96*	22.10±4.79*

* *indicated the significance level at p<0.5 as compared with LPS induced cells*

## Discussion

Inflammation is related with the pathophysiology of different medical conditions for example, joint inflammation and osteoarthritis. Non-steroidal anti-inflammatory drugs (NSAIDs) is one of the most well-known medications used worldwide^21^. The use of plants that have anti-inflammatory effects without side effects can be a good substitute for this drug because, in addition to their excellent anti-inflammatory properties, NSAIDs also have serious adverse effects such as perforation, GI ulcers, bleeding, and blockage^22^-^24^. In order to evaluate the anti-inflammatory potential of pomegranate peel extract, which is used in Indian traditional medicine, the current study was conducted.

Pomegranates, which were first grown in the Middle East, are today cultivated and grown all over the world. Currently, as a type of fruit high in polyphenols, it possesses anti-inflammatory, anti-diabetic, anti-microbial, anti-obesity, anti-cancer, and hypolipidemic effects^25^-^27^. Phenolic compounds are the extraordinary and significant components of pomegranate fruits, especially rich in the peel^28^-^30^.

Inflammatory response plays an important role in both normal physiology and pathology^31^. Various inflammatory mediators such as prostaglandin E2 (PGE2), nitric oxide (NO) and proinflammatory cytokines like TNF-α, IL-β and IL-6 are produced by the activation of the inflammatory cascade. One of the most important features in the inflammatory process is the production of free radicles such as reactive oxygen species (ROS) derived by phagocytic leucocytes. These free radicles produced as by products have the potential to cause oxidative stress in the cells leading to inflammatory and deleterious events. Studies have shown that phenolic antioxidants are able to prevent the induction of inflammatory cytokines by preventing NF-kB activation^32^,^33^ and the production of intracellular free radical species (ROS genration)^34^. The nuclear factor-kappa B (NF-kB) pathway has been identified as the target in inhibition of inflammation and serves as an important target drug development^35^.

In this study, the anti-inflammatory effects of Pomegranate peel extracts (PPM) were investigated using THP-1 cells stimulated with LPS. The cytotoxic effects of PPM on THP-1 cells are presented in figure 1. No cytotoxic effects were observed in THP-1 cells treated for 24hrs with PPM. The maximum reduction in cell viability was seen at a concentration of 400µg/ml.

Studies showed that ethanolic and methanolic extracts of Pomegranate aril have good capacity for anti-inflammatory activity^36^. The synthesis of macrophage nitric oxide synthase enzyme which generates nitric oxide is harmful to the affected tissue and many lead to acute or chronic inflammation^37^. Although several studies have been published on the pomegranate fruit peel waste^6^,^7^,^42^, there are still little published data on the effects of PPM n inflammation.

COX-2 is an enzyme that produces prostaglandins and is activated by LPS and other activators, including proinflammatory cytokines^38^. Our study showed that PPM suppresses the levels of COX-2 production in THP-1 cells but decrease in the expression of COX-2 was not observed upon pre-treatment with Quercetin. Findings have reported that pomegranate peel extract mediated suppression of COX-2 in chemically induced mammary carcinogenesis in rats. Several pomegranate constituents, example- ellagic acid, gallic acid and punicalagin A and B, inhibited lipopolysaccharide induced PGE2, nitric oxide and IL-6 production^39^.

Research studies stated that COX and lipoxygenase (LOX) which are key enzymes in the conversion of arachidonic acid to prostaglandins and leukotrienes (important inflammatory mediators), are inhibited by Punica granatum^40^, ^41^.

TNF is an efficient juxtracrine, paracrine and endocrine mediator of inflammatory and immune functions. It regulates the growth and differentiation of a variety of cell types^42^. Blocking NF-kb, inflammatory cell signalling pathways that produces various destructive factors may be a potential strategy to prevent inflammation.Our study did confirm that TNF-α expression of LPS-induced cells was affected after treatment with methanolic extract of pomegranate peel (PPM) and Quercetin. A decrease in TNF-α production was seen. IL-6 is one of the important pro-inflammatory factors, especially in early phage of inflammation. As shown in Table 4, decrease in the production of IL-6 was seen upon pre-treatment of the cells with the pomegranate peel extract. Research data suggested that quercetin might exert its anti-inflammatory effect through negatively modulating pro-inflammatory factors, such as IL-6. The inhibitory effect of the extract on pro-inflammatory factors production may provide a theoretical source on upcoming treatment of inflammation^43^.

Finally, our findings indicate that no cytotoxicity was observed after the treatment of THP-1 cells with pomegranate peel extract (25-400 µg/ml), but at higher concentration of 400 µg/ml, the cell viability decreased to 84% and attenuated the expression level of inflammatory cytokines COX-2 and TNF-α.

## Figures and Tables

**Table 1:** The IC50 values of the test samples after 24-hour treatment on THP-1 cells

Sample name	THP-1 cell line IC50 24hr
**Cisplatin**	9.46±3.49 µg/ml
**Quercetin**	NA (High cytotoxicity)
**PPM**	NA (Non-toxic)

**Abbreviations:** PPM= Methanol extract of Pomegranate peel
